# The geological origins and soil properties of loess-like silty clay: a case study in the jinan area

**DOI:** 10.1038/s41598-024-63394-0

**Published:** 2024-06-01

**Authors:** Zhenghao Liu, Xianfeng Ma, Dingyi Zhou, Linhai Lu, Haihua Zhang, Yujie Bai, Han Han

**Affiliations:** 1 Department of Geotechnical Engineering, Tongji University, Shanghai, 200092 China; 2 Jinan Rail Transit Group Co.Ltd., Jinan, 250101 China

**Keywords:** Loess-like silty clay, Geological genesis, Soil science characteristics, Middle and lower yellow river region, Engineering geology, Sedimentology, Stratigraphy

## Abstract

This study, using Jinan as a case study, systematically investigates the characteristics and geological genesis of loess-like silty clay in the middle and lower reaches of the Yellow River. The primary distribution of loess-like silty clay is revealed through field surveys, laboratory experiments, and previous literature reviews. The chemical and physical properties of the loess-like silty clay were examined, in addition to investigations into its mineral composition, microstructural characteristics, and engineering mechanical properties, in order to enhance comprehension of its attributes and formation mechanisms. The research suggests that the distinctive soil environment in the area has been influenced by numerous instances of the Yellow River overflow and channel shifts over its history, as well as the impacts of climate change, geological factors, and human activities. The primary sources of material for the loess-like silty clay consist of loess, Hipparion Red Clay, and paleosol layers. The discussion also addresses the impact of regional climate on the formation of mineral components. The aforementioned findings hold significant implications for advancing the understanding of historical climatic and paleogeographic shifts, as well as for addressing engineering challenges associated with the distribution of loess-like silty clay.

## Introduction

Loess-like silty clay, a distinctive type of soil, is especially abundant in the middle and lower sections of the Yellow River. This soil exhibits loess characteristics but differs from typical loess in terms of grain size distribution, composition, or genesis. Loess is primarily composed of silt, along with a certain proportion of fine sand, very fine sand, and clay particles. However, unlike typical loess, loess-like silty clay is a type of clayey soil with a plasticity index ranging between 10 and 17, and it has a high content of clay particles^1,2^. Loess-like silty clay and loess share the same color, which can easily lead to confusion. The development of loess-like silty clay in the Yellow River basin can be attributed to historical processes involving impact, erosion, and deposition by the Yellow River, which have evolved over millions of years. During the glacial periods, the extensive Loess Plateau and other areas underwent significant wind erosion, resulting in the formation of substantial amounts of fine-grained materials. The materials mentioned above were subsequently carried by wind and deposited at a considerable distance from their original location^3–6^. The Yellow River, functioning as a substantial natural conveyor, not only transported a considerable volume of sediment but also shaped distinctive alluvial plains in its middle and lower reaches^7–11^.

The advancement of urbanization in the Yellow River basin and other regions with comparable soils underscores the growing significance of investigating the soil properties of loess-like silty clay and its implications for engineering construction. The response of soil stability and erodibility is influenced by the particle composition, pore structure, and mineral content of the soil, in relation to moisture and pressure, as indicated by previous studies^12–16^. Addressing the physical properties and engineering characteristics of loess-like silty clay, Zhang et al*.*^17^ performed cone penetration tests on the loess-like muddy silty clay in western Henan and developed an empirical model for the physical and mechanical parameters of loess-like mud-rich cohesive soil. Huang et al*.*^2^ conducted unsaturated triaxial tests on loess-like silty clay in the Sanmenxia region, examining the changes in matric suction of the loess-like silty clay. Miao et al*.*^18^conducted an inversion analysis on the subgrade reaction coefficients of loess-like silty clay in the Shijiazhuang area, resulting in the derivation of theoretical calculation reference formulas. Al-Harthi^19^ identified a correlation between land subsidence and ground fissures in the Wadi Al-Lith region of western Saudi Arabia and the rapid decline in groundwater levels subsequent to flooding, as well as the hydro-consolidation of loess-like silty clay. The results of their X-ray diffraction analysis indicated that the clay is mainly composed of kaolinite and illite, with a minor presence of smectite, though without in-depth analysis its origins. However, despite these advances, comprehensive studies on the mineral content, particle composition, and pore structure of loess-like silty clay, as well as its origins and connections with loess, remain limited.

Given the current paucity of research on loess-like silty clay internationally, the extensive presence of this unique soil type has been unveiled through construction projects in the middle and lower reaches of the Yellow River in China, especially in the Jinan area. We have conducted substantial geological surveys and analyses, providing a wealth of data on loess-like silty clay, thus enriching the field and offering parameters for the study of this distinctive soil. After conducting an extensive literature review and experimental analyses, it has been established that loess should be regarded as a primary source of the loess-like silty clay. Indeed, as research on loess advances, the international community is increasingly recognizing the ubiquity of loess research. From China to regions such as the Middle East, Central Asia, and even North America, the widespread occurrence of loess deposits is being reported. Despite China's location on the eastern side of the Eurasian continent, its Quaternary period environmental evolution exhibits regional characteristics and patterns yet is also governed by universal global laws. This suggests that during the Quaternary period, especially in the middle and lower reaches of loess deposit regions, a substantial presence of loess-like silty clay is likely. This implies that our research findings are relevant not only to China but also to many regions worldwide with loess deposits, as illustrated by Al-Harthi, AA's study in the Al-Ula area of western Saudi Arabia. This further indicates the global potential presence of this special soil, especially in regions with loess deposits, highlighting the necessity of research into the distribution, formation, physicochemical properties, and engineering behavior of loess-like silty clay, which will provide a reference for future engineering practices in more extensive regions globally and further international interest and understanding of this unique soil.

## Spatial distribution and geological origins of loess-like silty clay in the yellow river's middle and lower region

**Figure 1:**
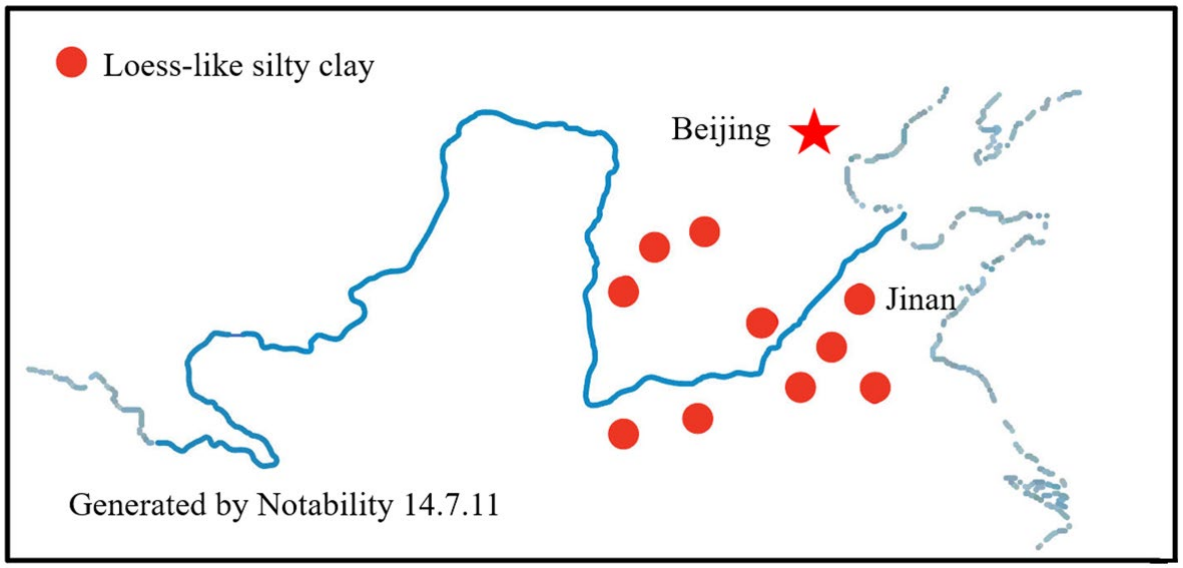
Distribution of silty clay in the Yellow River basins. The map illustrates the regional distribution of silty clay in the Yellow River basin in China. The red dots on the map indicate regions with loess-like silty clay distribution. The map is drawn using Notability 14.7.11, linked at https://apps.apple.com/cn/app/notability-%E7%AC%94%E8%AE%B0-pdf/id360593530, and modified using PowerPoint 2016.

Loess-like silty clay is primarily distributed in the middle and lower regions of the Yellow River basin in terms of spatial distribution, as illustrated in Fig. 1. Significant occurrences of loess-like silty clay strata have been identified in various regions, including most of Henan Province, the central and southern parts of Shanxi Province, the southern region of Hebei Province, the eastern area of Shandong Province, the northern sections of Anhui Province, and the northern parts of Jiangsu Province. The mentioned regions are primarily situated in temperate monsoon and semi-arid climates of inland areas, where the accumulation of loess-like silty clay is mainly driven by river transport and seasonal wind activity.

**Figure 2:**
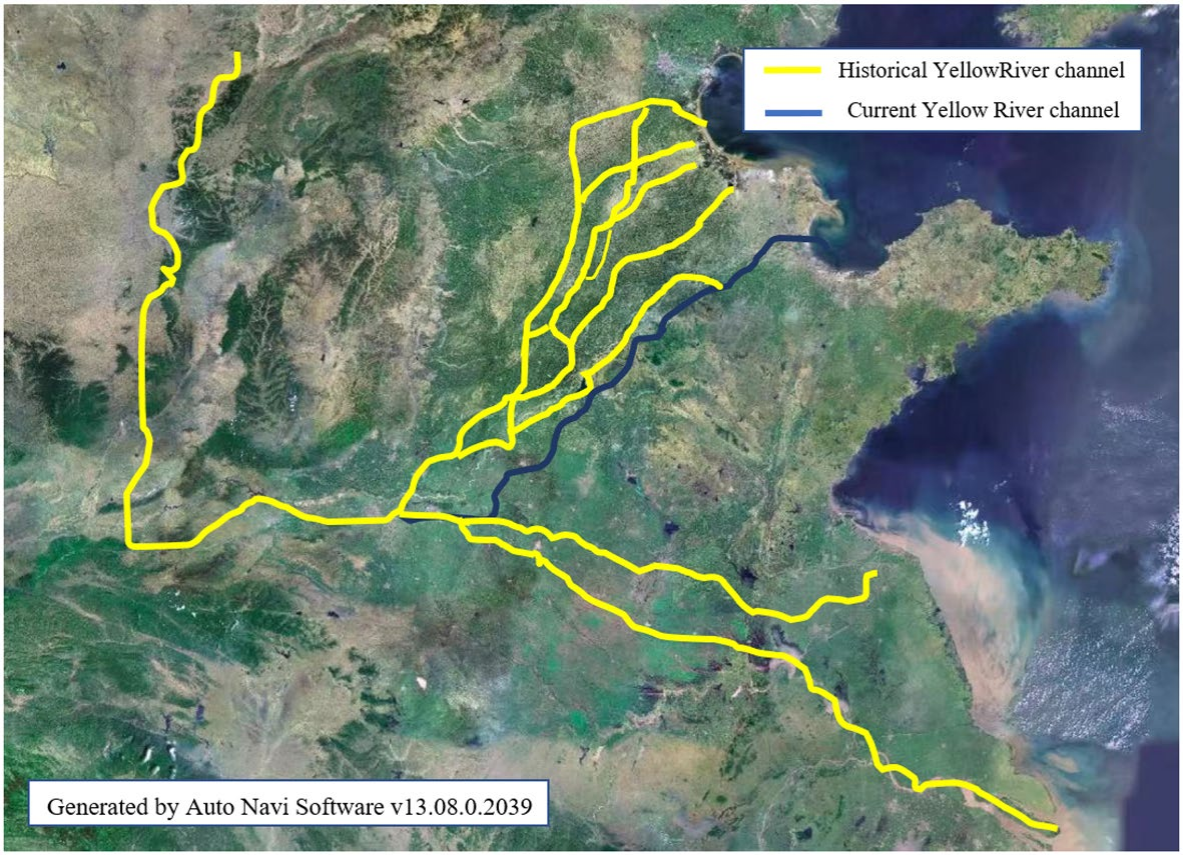
Major historical channels of the Yellow River. The map illustrates the main river channels of the Yellow River throughout its history compared to the present-day course. The yellow lines represent the historical river channels of the Yellow River, while the blue lines depict the current river channel. The map is drawn using AutoNavi Software v13.08.0.2039, linked at https://ditu.gaode.com/, and modified using PowerPoint 2016.

The distribution of loess-like silty clay in the middle and lower reaches of the Yellow River basin is intricately linked to the historical evolution of the Yellow River. The Yellow River, renowned for its significant sediment load, has experienced numerous alterations in its course and breaches as a result of natural calamities and human interventions. Historical records have indicated that from the year 602 AD, the Yellow River has experienced over 1500 instances of breaching its banks and has undergone significant changes in its course more than 70 time^20–22^. The primary historical channels are depicted in Fig. 2.

The alteration of the Yellow River's path frequently leads to extensive flood calamities. During flood events, substantial volumes of loess-like silty clay were transported to regions bordering rivers, where they accumulate around the riverbanks, giving rise to floodplains and alluvial plains. Using the Shandong Province as a case study, the climate patterns across Asia during the Upper Pleistocene were significantly impacted by the continuous uplift of the Tibetan Plateau, while the crustal subsidence resulting from oscillatory movements in the North China region created favorable conditions for sedimentary processes^23–25^.

The global climate during the Upper Pleistocene underwent multiple cycles of glacial and interglacial periods. During glacial periods, various factors including decreased sea levels, heightened river gradients, and diminished vegetation resulted in intensified river erosion, resulting in the transportation of significant sediment loads in the Yellow River. Conversely, in interglacial periods, the rising sea levels led to an increase in the sedimentary activity of the river. The Yellow River, carrying sediments from the Loess Plateau, initiated the deposition of sediment layers upon entering the level terrains of Shandong Province. This process resulted in the gradual formation of a stratum approximately 40–50 m in thickness.

Jinan, straddling the north and south banks of the Yellow River, is one of the largest and most important centre cities in the middle and lower reaches of the river. The unique geographical and historical context of Jinan, combined with the northward urban expansion and the increase in engineering projects along the riverbanks in recent years, has led to the widespread discovery of loess-like silty clay in the area. This discovery makes Jinan an ideal case study for investigating the role and impact of this soil type in urbanization, especially in engineering construction. The urban area of Jinan exhibits a piedmont alluvial fan geomorphology, featuring relatively low terrain and a flat topography. Based on the geomorphological features of the Jinan area, it can be categorized into two Level I geomorphic regions: plains and mountains. Based on their origin and physical characteristics, they are further categorized into Level II regions, as illustrated in Table 1. Under the influence of concentrated surface water flows, loess-like silty clay gives rise to gullies of various shapes, such as broad gullies, wedge gullies, lane gullies, and small erosional gullies.

**Table 1:** Geomorphological classification.

Level I		Level II	Micro-geomorphology
Plain Geomorphology	Accumulative Geomorphology	Alluvial Plain	Floodplains, artificial levees, swamps, fishponds
		Fluvial-Alluvial Plain	Riverbeds, terraces, gullies, embankments
Mountainous Geomorphology	Erosional Geomorphology	Residual Hills and Hillocks	Gullies, "U"-shaped valleys, terraces, cliffs, landslides
		Low Mountains	"V"-shaped valleys, man-made cliffs, landslides
	Karst Geomorphology	Karstified Hills and Low Mountains	Caves, karren, solution channels

Furthermore, human agricultural practices, construction activities, and land development can impact the distribution of loess-like silty clay, potentially leading to its expansion or reduction in range. This requires extensive geological surveys carried out collaboratively by multiple departments. We acknowledge that these interventions predominantly exhibit localized characteristics, these changes are spatially limited and do not fundamentally alter the overarching distribution pattern of loess-like silty clay across the Yellow River basin.

## Materials and results

The material properties of soil are influenced by geological processes, which subsequently impact the compositional and structural characteristics of the soil. The compositional and structural characteristics mentioned above delineate the physical properties of the soil. These properties, in conjunction with stress conditions, govern the engineering properties of the soil. Consequently, a comprehensive understanding of this special soil requires a multifaceted and multidimensional analysis. It is essential to elucidate its physicochemical properties, particle and mineral composition, and to explore its distinctions and connections with loess.

### Physicochemical properties and particle composition

**Figure 3:**
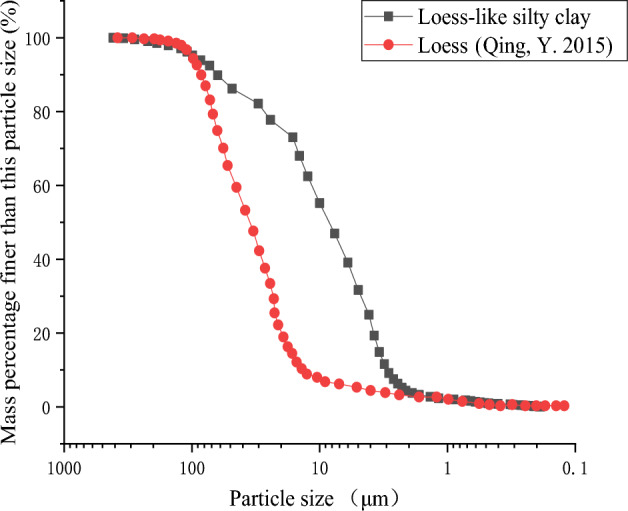
Particle size classification of silty clay.

The soil used for testing is the 12th layer of loess-like silty clay from Jinan City, Shandong, as specified in the "Geotechnical Engineering Exploration Stratum Sequence Division Standards for Urban Area of Jinan"^26^. The undisturbed soil exhibits a yellow–brown colouration and possesses a natural dry density of 1.62 g/cm^3^. The specific gravity of the soil is 2.73, with a liquid limit of 27.6%, a plastic limit of 17.2%, and a plasticity index of 10.4. Following the directives of TB 10103—2008, “Regulations for Rock and Soil Chemical Analysis of Railway Engineering”, experiments were conducted on samples obtained from boreholes at five distinct depths, with the results averaged^27^. The test results for the chemical properties of the soil are presented in Table 2. Particle size tests were conducted using the Malvern Master Sizer 3000 Laser Particle Analyzer. Figure 3 depicts the classification and composition of soil particle sizes.

**Table 2:** Soil chemical properties test results.

Name	Content	Unit	Name	Content	Unit
Na^+^	165.6	mg/kg	HCO_3_^−^	249.14	mg/kg
K^+^	19.2	mg/kg	CO_3_^2−^	76.24	mg/kg
Ca^2+^	185.07	mg/kg	OH^-^	Not detected	mg/kg
Mg^2+^	20.64	mg/kg	CEC	13	cmol/kg
Cl^-^	39.5	mg/kg	pH	7.9	–
SO_4_^2−^	60.1	mg/kg	Soluble salt content	0.93	g/kg

The analysis of the soil samples reveals that the silty clay, similar to loess, has a pH of 7.9, demonstrating slight alkalinity. Unlike loess, which is rich in soluble salts, this soil has a low soluble salt content of 0.93 g/kg, categorizing it as low-salt soil. Its cation exchange capacity of 13 cmol/kg suggests a moderate ability to hold water, along with moderate plasticity and shrinkage potential. The analysis of ion content shows the presence of sodium, potassium, calcium, magnesium, and sulfate ions, with notable levels of calcium and sodium ions, a characteristic similar to the calcium-rich nature of loess^28^. Sodium ions, due to their substantial hydration effect, can attract a greater number of water molecules, resulting in the formation of larger hydrated ions. This phenomenon may result in heightened electrostatic repulsion at the clay particle surface, leading to a decrease in the cohesive forces between particles and subsequently causing instability in soil structure upon moisture exposure. Moreover, the area experiences a continental monsoon climate, characterized by annual precipitation of 648 mm, evaporation of 2263.00 mm, and interannual variability in runoff, marked by different wet and dry seasons. This cyclic fluctuation in moisture levels has the potential to induce geological issues, including landslides, ground subsidence, and cracking.

The particle analysis findings reveal that the examined loess-like silty clay predominantly consists of silt and clay particles, with the 0.05 ~ 0.005 mm particle size range being the predominant component, constituting 54.55% of the overall content. The group with particle sizes ranging from 0.5 ~ 0.25 mm comprises 0.94% of the total, while the 0.25 ~ 0.075 mm and 0.075 ~ 0.05 mm groups represent 6.61% and 6.24% respectively. Additionally, particles smaller than 0.005 mm make up 31.66% of the total. When compared to typical loess^29^, this loess-like silty clay shows a distinct difference in the proportion of silt and clay particles, particularly in the 0.002–0.2 mm range, where the loess-like silty clay has a significantly higher percentage of silt and clay particles than standard loess.

### Mineralogical composition analysis

Clay minerals constitute the primary components of silty clay and play a significant role in controlling the engineering geological properties and variations of cohesive soils. The Japan Rigaku D/max2500 X-Ray Diffractometer was employed in this study to qualitatively identify and quantitatively analyze the mineral composition present in both the coarse and fine particles of loess-like silty clay sourced from the Jinan area. The loess-like silty clay samples were pulverized to a particle size of less than 1 mm. Subsequently, centrifugal separation techniques were utilized to extract particles within the size ranges of 20-63μm (coarse particle) and less than 2μm (fine particle) for X-ray diffraction analysis. This analysis encompassed samples from five different locations at varying depths, totaling ten sets.

Loess-like silty clay comprises a combination of detrital minerals and clay minerals. The coarse grain fraction, predominantly ranging from 0.1 to 0.01 mm in diameter, forms a substantial part of the silty clay's composition. The primary constituents of coarse particles consist of quartz, microcline, and plagioclase, along with a minor presence of calcite and mica. The specific contents are presented in Table 3.

**Table 3:** Coarse particle mineral composition.

Mineral composition	Quartz(%)	Plagioclase feldspar(%)	Orthoclase feldspar(%)	Calcite(%)	Mica(%)	pH value
Average value	68	12	10	6	3	8.0
Range value	64 ~ 72	10 ~ 14	8 ~ 12	5 ~ 7	2 ~ 4	7.9 ~ 8.1

Clay minerals constitute the predominant composition of loess-like silty clay and exert the most significant influence on its reactivity. In this study, the composition of clay minerals in particles smaller than 0.005 mm was extracted from the loess-like silty clay and subsequently analyzed. The experimental results are presented in Table 4.

**Table 4:** Fine particle mineral composition.

Mineral composition	Illite (%)	Montmorillonite (%)	Chlorite (%)	Kaolinite (%)	Vermiculite (%)	pH value
average value	56	23	10	8	3	8.0
range value	52 ~ 60	21 ~ 25	8 ~ 12	6 ~ 10	2 ~ 4	7.9 ~ 8.1

The primary mineral constituents of the fine clay particles in loess-like silty clay consist of illite, montmorillonite, chlorite, kaolinite, and vermiculite. Comparison with loess reveals that both loess-like silty clay and loess share a similar mineral composition, particularly the presence of calcite, indicating a rich calcium content that underscores their connection^30^. The hydrophilic, adsorption characteristics, and ion exchange capacities of the minerals mentioned above have a substantial influence on the engineering geological properties of the silty clay. Illite is clearly the predominant component found in loess-like silty clay, which is characterized by a relatively moderate surface area, ion exchange capacity, and a medium-thick diffuse double layer. The mentioned attributes align with the genuine engineering geological traits of loess-like silty clay, including moderate moisture content, Atterberg limits, swelling-shrinking tendencies, and moderate mechanical strength.

### Microstructure of soil particles

The microstructure of loess-like silty clay plays a significant role in determining its engineering geological characteristics. This study used Scanning Electron Microscopy (SEM) and Energy Dispersive X-ray Spectroscopy (EDS) with a Hitachi Regulus 8100 field emission microscope for detailed analysis. Five sample groups from different sampling locations were examined.

**Figure 4:**
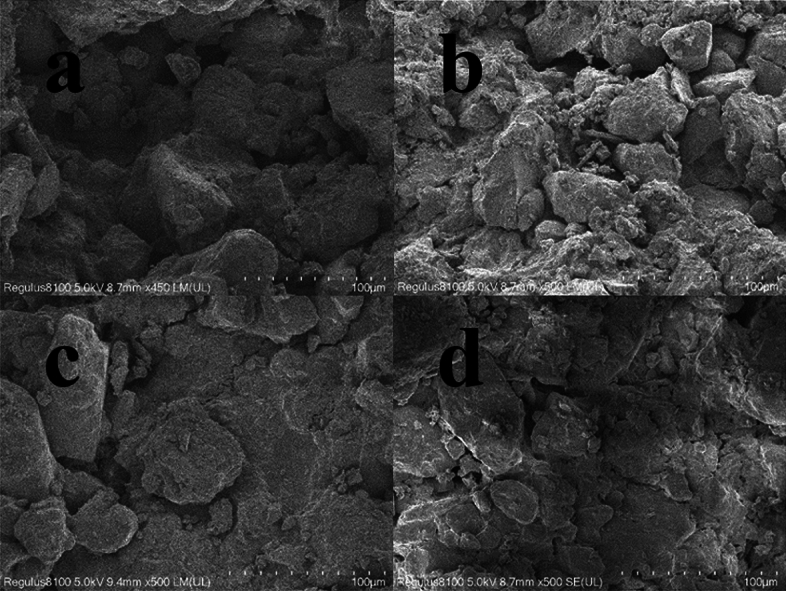
Framework structure of loess-like silty clay.

The analysis of SEM images indicates that the microstructure of the loess-like silty clay conforms to a framework type, as illustrated in Fig. 4. The framework particles exhibit a wide range of sizes, including sand particles larger than 0.05 mm, coarse silt-sized debris ranging between 0.01 and 0.05 mm, and fine silt and clay particles. The substances mentioned above combine and solidify to create aggregates, which serve as the main structural components of loess-like silty clay. While loess-like silty clays typically display homogeneity, the specific loess-like silty clay under investigation demonstrates a certain degree of heterogeneity on a smaller scale. Certain regions exhibit high density, whereas others display significant porosity. This relates to the basic structural units of loess—the granular and agglomerate units and their connection methods—contact and cementation connections, which have similarities^28^.

**Figure 5:**
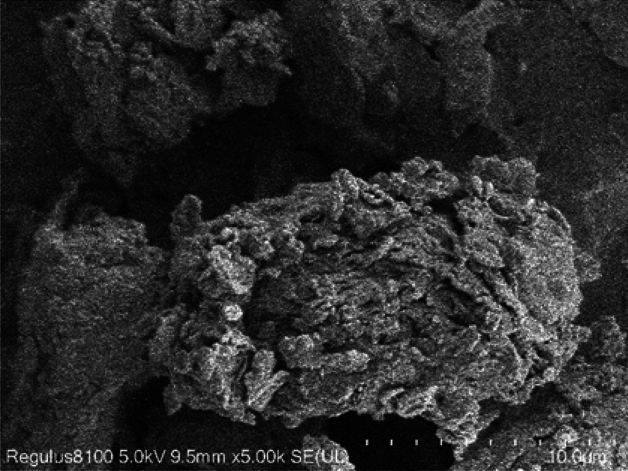
Clay particles. The figure shows an aggregate composed entirely of clay particles. This is an unique combination formed through the cementing action of clay, facilitating the connection of framework particles.

Unlike loess, although most of the clumps are composed of silt and clay particles, a distinct subset is formed entirely of clay particles, as illustrated in Fig. 5. The distinctive combination is enabled by the cementation of the clay, which enhances the bonding between the framework particles.

**Figure 6:**
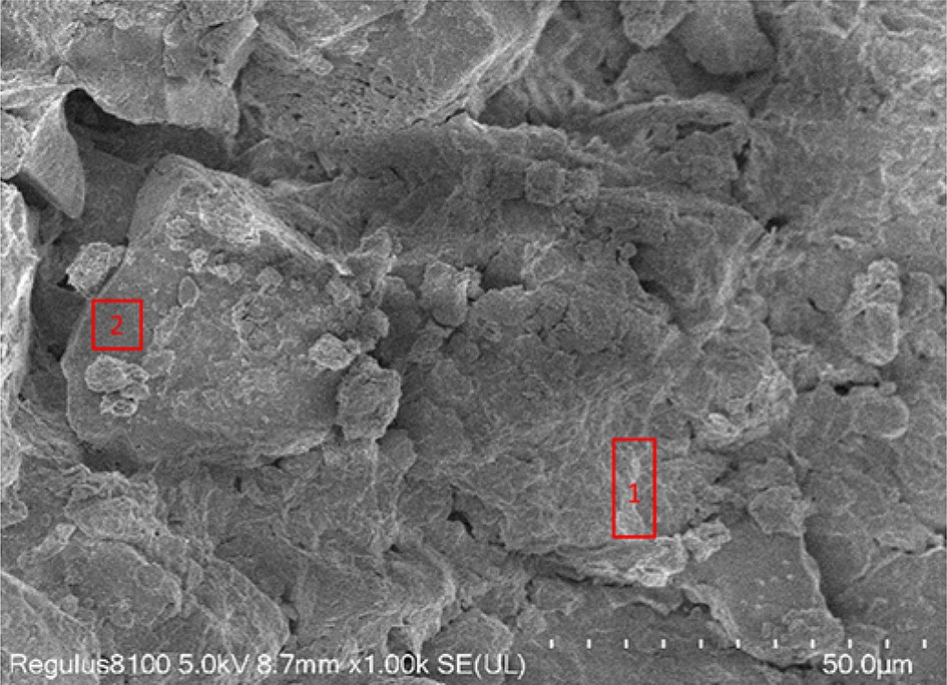
Chemical element analysis using the EDS system. The electron microscope scanning results in the figure depict the cementing material on the surfaces of the clastic particles of loess-like silty clay. Area 1 in the image represents a clearly flocculent encasement bonding region, while Area 2 corresponds to the particle region.

Additionally, it seems that the majority of the fragmented particles within the primary mass of silty clay resembling loess are enveloped by a slender coating of binding substance, establishing structural connections among the particles of silty clay resembling loess. The integrated EDS system of SEM allows for the analysis of elemental composition in specific areas. Through this approach, it is possible to conduct microanalysis of the elemental composition of the cementing substances and particles in SEM images of the silty clay. In Fig. 6, Region 1 represents a distinct flocculent cementing area, while Region 2 corresponds to the particle area. The elemental composition analysis results of the cementing material and particles are displayed in Table 5.

**Table 5:** Elemental composition analysis of different areas within loess-like silty clay.

Analysis location	Main elements analysis results	Inferred COMPONENTS
Cementing material	O, Al, Si, C, Ca	Calcareous cement
Particles	O, Na, Al, Si	Quartz, Feldspar (Silicates)

The analysis presented in Table 5 demonstrates that the predominant constituents of the cementing material include oxygen (O), aluminum (Al), silicon (Si), and calcium (Ca). Upon analysis of the mineral composition of the silty clay, it can be inferred that the cementing material is predominantly composed of a flocculent calcareous substance resulting from the inter-cementation of calcium carbonate and montmorillonite. The predominant elements found in soil particles include O, Na, Al, and Si, with the primary minerals consisting of quartz and feldspar, both of which are silicate minerals. The calcium content in the cementing material exceeds that found in the particle areas to a significant degree.

The pore space within the framework structure of loess-like soils can be classified into three primary types, as evident from the analysis: Contact pores exist within the framework, where the particles are in close contact with no space for deformation; Cemented contact pores refer to the consolidation of framework particles through the presence of clay minerals and calcareous cement, resulting in the development of specific structural strength; Isolated pores are present, in which the framework particles are in point-contact with each other, and are accompanied by pores of comparable size to the framework particles themselves. The quantity, distribution, and presence of calcareous cementing material have a significant impact on the mechanical properties of loess-like silty clay, playing a crucial role in determining its behavior.

### Engineering mechanical characteristics

The formation process of loess-like silty clay is characterized by complexity, uncertainty, and variability, which in turn affect its engineering properties. In this study, more than 10 observation points were utilized, and over 1,000 sets of experimental data on loess-like silty clay were compiled to investigate distribution characteristics and associated patterns in greater detail. The statistical findings are displayed in Table 6.

**Table 6:** Statistical summary of physical and mechanical indices of loess-like silty clay in Jinan area.

Indicator	Sample size	Distribution range	Mean	Standard deviation	Variance	Coefficient of variation
Moisture content W/$\%$	1011	16.1–39	25.87	2.698	7.278	0.104
Wet density/$\text{g}/{cm}^{3}$	931	0.95–2.08	1.935	0.076	0.0058	0.039
Void ratio	931	0.59–1.12	0.773	0.107	0.0115	0.138
Saturation/$\%$	931	65–100	91.62	6.149	37.81	0.067
Liquid limit index	1011	-0.16–1.07	0.427	0.1775	0.0315	0.416
Liquid limit/$\%$	1011	24.4–44.9	33.982	3.904	15.243	0.115
Plastic limit/$\%$	1010	15.2–35.5	20.14	2.216	4.914	0.110
Plastic index	1011	5.5–21	13.84	1.864	3.474	0.135
Compression ratio/${\text{MPa}}^{-1}$	873	0.09–0.82	0.2445	0.31	0.0974	1.268
Bulk modulus/$\text{MPa}$	873	2.2–18.6	1.857	0.316	0.10	0.170

The analysis of the statistical data presented in Table 6 indicates a variability in the indices of loess-like silty clay in the Jinan area. The coefficients of variation for wet density and saturation exhibit relatively small values. The mean saturation of loess-like silty clay is 91.61%, with a coefficient of variation of ≤ 0.07, suggesting that the loess-like silty clay in the Jinan area is predominantly in a saturated state. Water content, porosity ratio, liquid limit, plastic limit, and plasticity index, as well as the modulus of compressibility, demonstrate average variability. The void ratio of loess-like silty clay is similar to that of loess; however, its liquid limit, plastic limit, natural moisture content, and saturation are all higher than those observed in loess^31^. The integration of mineralogical composition and Scanning Electron Microscope (SEM) examination results indicates a higher content of clay minerals within loess-like silty clay. These clay minerals exhibit significant cohesiveness, surface energy, and hydrophilicity, which not only strengthen the interparticle binding force but also markedly enhance the soil's water retention capacity.

In contrast, the coefficients of compression and liquidity index display high variability. The compression coefficient generally falls below 0.6 MPa^-1^, averaging at 0.25 MPa^-1^. In certain areas, it exhibits high compressibility. The substantial variability of the liquidity index suggests regional disparities in the plasticity and flow characteristics of the soil. Therefore, it is important to take into account the variability of the compression coefficient and liquidity index when calculating engineering properties. Directly using average values is not recommended; instead, it is advisable to use a weighted average value that takes into account the variability of the indices. Based on our test results in sections "Physicochemical properties and particle composition" to "Microstructure of soil particles", the high variability in the liquid limit and compression coefficient of loess-like silty clay is primarily attributed to the complex interplay of soil mineral composition, microstructure, chemical properties, and environmental conditions. Chemical properties, including various concentrations of ions such as sodium, potassium, calcium, and others, contribute to this variability. Notably, calcium ions can emulate the calcareous nature of loess, influencing the soil's plasticity and compression behavior through calcareous cementation. The high hydration of sodium ions can increase the electrostatic repulsion among soil particles, thus affecting the soil's liquid limit and compression coefficient. The soil's mineral composition, including illite, montmorillonite, kaolinite, glauconite, and vermiculite, affects its water absorption capacity, and consequently its volume, plasticity, and compression behavior. The soil's heterogeneous microstructure impacts its engineering properties, where different types of cementation and particle contact methods affect compressibility. Environmental conditions, characterized by a continental monsoon climate with distinct wet and dry seasons, cause variations in moisture content, directly impacting soil plasticity and compressibility. The interplay of these factors results in the liquid limit and compression coefficient characteristics of loess-like silty clay.

These findings delineate both differences and connections between loess-like silty clay and loess. In terms of particle size distribution, the primary distinction lies in the content of clay and silt particles, with loess-like silty clay having a notably higher clay particle content than loess. Mineralogical composition analyses reveal that despite a consistency in mineralogical composition between loess and loess-like silty clay, especially the presence of calcite indicating abundant calcium content, this reflects their interrelation. The differences in particle composition, clay mineral content, and their skeletal structures further delineate the engineering property disparities between loess-like silty clay and loess.

## Discussion

### Provenance discussion of loess-like silty clay

The occurrence of suspended rivers in the lower Yellow River is mainly attributed to the sediment accumulation, a result of the significant load of silt and sand transported by the Yellow River. While there is ongoing academic discussion about the precise origins and distribution of the mentioned sediments, there is a widespread agreement that the Loess Plateau plays a substantial role in contributing to the sediment load of the Yellow River. Rivers play a pivotal role in the transportation process that contributes to the formation of silty clay resembling loess. Consequently, it is reasonable to infer that loess serves as one of the primary origins of loess-like silty clay.

In particular, there is a uniformity in the mineral composition of loess and silty clay; however, loess is primarily comprised of silt particles and contains fewer clay particles. However, the widespread occurrence of loess-like silty clay layers in many urban areas along the middle and lower sections of the Yellow River cannot be exclusively ascribed to the deposition of clay particles derived from loess and transported by the river. Consequently, the development of silty clay with loess-like characteristics is not solely attributed to the loess transported by the Yellow River, but also has a direct correlation with the geological sediments of the Loess Plateau.

During experimental investigations, it was observed that loess-like silty clay, which appears yellow–brown when dry, turns reddish upon oxidation in a moist environment. Unlike synchrotron radiation X-ray diffraction (XRD), conventional XRD cannot detect iron oxides^31^. Our analysis of five samples using synchrotron radiation XRD indicated the presence of iron oxides with low crystallinity in loess-like silty clay. There is a weak diffraction peak at 0.242 nm, providing a direct scientific basis for our discussion on the characteristics of iron ions, even though these data were not directly reflected in Tables 2 and 4. Owing to its slightly alkaline pH, the iron primarily exists in an insoluble inorganic state, leading to limited reactive iron and influencing the mobility of iron in the soil. Under arid conditions, the reactivity of iron oxides with water is limited, resulting in the soil retaining its yellow color in a relatively constant state. However, when the soil becomes moist and undergoes oxidation, the Fe^2+^ in the iron minerals undergoes oxidation to Fe^3+^, resulting in the formation of new crystalline structures that modify the soil's light absorption and reflection properties. This alteration causes the moist soil to exhibit a reddish-brown appearance^32^.

The loess strata are known for containing the most comprehensive and complete geological information dating back 2.5 million years. Further investigation into the Quaternary of the Loess Plateau is required to examine the origins of iron oxides and clay particles. Taking the Luochuan loess profile as a case study, the Luochuan area commenced the accumulation of approximately 20 m of thick Hipparion Red Clay (Tertiary Red Beds) by the conclusion of the Pliocene. Studies have indicated that this clay contains high proportions of clay particles (37–55%), as well as substantial quantities of montmorillonite and illite, which are expansive clay minerals exhibiting robust physicochemical activity. A 135 m-thick layer of loess is deposited on top of the red clay, consisting of alternating loess layers, paleosols, and weathered loess layers. The paleosols display ferruginization, frequently manifesting as reddish-brown or brownish-red, and containing microstructures of optically oriented clays that have been documented. This indicates that the paleosols found in the loess and the Hipparion Red Clay are probable substantial contributors of iron oxides and clay particles.

Sun et al.^33^ conducted a study on paleosols intercalated within loess layers. They gathered multiple paleosol samples from the Liujiapo loess profile near Xi'an for analysis of granulometry and mineralogical composition. The clay particle content in the previously mentioned paleosols was found to be over 60%, as indicated in Table 7. Liu et al*.*^34^ conducted an analysis of the mineralogical characteristics of different paleosol layers, and the findings are detailed in Table 8.

**Table 7:** Particle size content of paleosols.

Stratigraphic position	Soil type	Particle size content (%)				
		> 0.05 mm	0.05–0.01 mm	0.01–0.005 mm	< 0.005 mm	< 0.002 mm
Holocene	Paleosol	0.5	25.0	11.1	63.4	53.2
Upper lishi loess	Paleosol	0.7	24.4	9.8	65.2	55.8
Lower lishi loess	Paleosol	0.6	23.6	11.2	64.6	53.8
Wucheng loess	Paleosol	0.3	21.1	11.1	67.7	55.2
Lantian formation	Red clay	0.5	15.0	8.0	76.5	63.0

**Table 8:** Overview of mineralogical components and characteristics in loess paleosols.

Loess paleosol layer	Mineral composition
Holocene	Illite, Montmorillonite, Kaolinite, Quartz, Calcite, Organic Matter
Upper pleistocene	Illite, Montmorillonite, Kaolinite, Glauconite, Calcite, Quartz
Middle pleistocene	Illite, Montmorillonite, Kaolinite, Montmorillonite, Quartz, Fe_2_O_3_

The analysis presented in Table 8 demonstrates that the main mineral constituents of the paleosols found in the loess are largely similar to the silty clay minerals. The unique coloration of paleosols found in loess deposits, which is defined by a high colloidal particle content, closely resembles that of silty clays. The substantial thickness and similar mineral composition, in conjunction with the aforementioned attributes, strongly indicate that paleosols are a significant source material for the formation of silty clay.

Hipparion Red Clay, is characterized by its striking purple-red, brick-red, and yellow–brown coloration. It is extensively distributed in the middle and upper reaches of the Yellow River region. The red color of this clay is attributed to its iron oxide or hydroxide content. The differences in red and yellow shades are indicative of the diverse climatic conditions during deposition and the varying proportions of Fe^2+^ to Fe^3+^ ions, which contribute to the soil's distinct colors. Following the diagenetic process of the Hipparion Red Clay, natural processes such as weathering, unloading, and shrinkage played a significant role in the formation of various jointing and fracture patterns, including structural and weathering joints. The previously mentioned changes have weakened the clay's ability to resist erosive and deflationary forces. Moreover, the soil's high salinity and dispersivity increase its susceptibility to generating substantial solid runoff when exposed to rainwater. The mentioned runoff materials are carried by the Yellow River, offering a substantial source of material for the downstream loess silty clay layers.

Li et al.^35^ conducted an analysis of the particle size and mineral composition of Hipparion Red Clay. Their findings revealed a predominance of clay particles, with the clay-size fraction (< 0.005 mm) typically ranging from 38 to 54%, as presented in Table 9.

**Table 9:** Granulometric composition (%) of Hipparion Red Clay.

Particle size range	> 0.075 mm	0.075 ~ 0.005 mm	< 0.005 mm	< 0.002 mm
Content %	0.08 ~ 3.41	45.02 ~ 62.71	37.12 ~ 54.40	30.24 ~ 48.04
Average %	1.54	55.2	43.3	37.1

The soil is primarily composed of minerals such as feldspar, quartz, and calcite in terms of its mineral content. The predominant clay minerals are primarily illite, with the presence of kaolinite and montmorillonite as well^28^. This composition is fundamentally in line with the mineral composition of loess-like silty clay. Given the erodibility of Hipparion Red Clay, its distinctive red hue, high concentration of clay-sized particles, and its mineral and chemical composition resembling that of loess-like silty clay, Hipparion Red Clay serves as a significant source material for silty clays.

In summary, the formation of loess-like silty clay is influenced by a multitude of factors and encompasses a complex process that incorporates contributions from various sources, including the loess from the Loess Plateau, paleosol layers, and Hipparion Red Clay.

### The impact of climate on mineral characteristics

Loess-like silty clays exhibit differences in both visual characteristics and mineral composition compared to those present in the Yangtze River and Pearl River basins. The differences mentioned above stem from diverse material sources and are also influenced by regional climatic conditions, especially fluctuations in temperature and humidity. The factors mentioned above play a crucial role in determining the formation and transformation of minerals^36,37^. Distinct climatic regions result in different weathering patterns and soil compositions, contributing to geographical differences in mineral compositions and formations, as illustrated in Table 10.

**Table 10:** Characteristics of silty clay in different climatic regions.

	Loess-like silty clay	Grey silty clay	Red silty clay
Characteristic	1. Exhibits a higher sand content, with an appearance that ranges from yellow–brown to yellowish-brown 2. Rich in quartz and feldspar, contains a significant amount of montmorillonite, with considerable calcite and minor amounts of amphibole; kaolinite is less common	1. Typically has a color spectrum ranging from grey to greyish-brown 2.Abundant in illite and kaolinite, with montmorillonite being relatively scarce	1. Coloration generally spans from red to reddish-brown 2. Enriched in kaolinite, with minimal montmorillonite content, and a certain presence of gibbsite and goethite

The sediment of the Yellow River originates mainly from the Loess Plateau and contains a high concentration of minerals, including quartz, feldspar, and mica. The arid and cold climate in this region restricts the scope of chemical weathering, while the alternating dry and wet climatic conditions promote the development and conservation of minerals such as calcite, montmorillonite, and glauconite, but are not favorable for the formation of kaolinite. The high calcium content of loess facilitates the development of calcite in alkaline or slightly alkaline conditions. Under soil conditions ranging from neutral to strongly alkaline, with minimal leaching, the potassium ions located within the layers of the illite lattice may undergo leaching and subsequently be substituted by magnesium ions. This process results in the conversion of illite into montmorillonite.

Conversely, the climatic conditions in the Yangtze River basin, characterized by elevated temperatures and increased precipitation, as well as the weakly acidic or acidic composition of the soils, enhance chemical weathering. This environment is conducive to the formation of kaolinite in warm and moist climates with sustained and vigorous hydrolysis. This process leads to a decrease in the montmorillonite content and an increase in the kaolinite content, as well as the leaching of alkaline earth metals such as Ca, resulting in reduced presence of calcite in the clay. Illite forms under less intense leaching conditions in continental, neutral, or slightly alkaline aqueous environments. As the climate becomes wetter and hotter, there is an intensification of chemical weathering and ion exchange, resulting in an increase in Al^3+^ ions within the illite. Furthermore, the progression of chemical weathering leads to the strengthening of hydrolysis, which further transforms illite into kaolinite^38^.

In the Pearl River basin, specifically in the Xijiang area, the warm and rainy climate has resulted in heightened chemical weathering processes. In this acidic soil environment, montmorillonite undergoes dissolution, leading to the abundant formation of kaolinite. This process is a consequence of extensive leaching in a humid climate under acidic conditions and can also involve the transformation of montmorillonite into kaolinite. Mica-type minerals may undergo sequential transitions from illite to montmorillonite and finally to kaolinite, as a result of varying degrees of weathering and leaching. This is associated with the appearance of iron and aluminum hydroxides, such as gibbsite and goethite. Alkali and alkaline earth metals, such as calcium, undergo significant leaching, resulting in the absence of calcite in the sediments of the Pearl River.

## Conclusion

Current research on loess-like silty clay is limited within the academic community, especially regarding its origins, mineral composition, structural characteristics, and engineering physical properties, with even fewer references available. This study focuses on the Jinan area as a case study, employing a combination of pedology and engineering geology techniques for the first systematic analysis of the geological genesis and pedological characteristics of loess-like silty clay in the middle and lower reaches of the Yellow River. The findings indicate that the extensively distributed loess-like silty clay in the region primarily arises from the prolonged erosion, transportation, and sedimentation processes of the Yellow River and its tributaries. Comparative analyses of mineral composition and particle size testing indicate that the reddish-brown earth of Hipparion, loess, and paleosol layers are recognized as the main sources of material for the silty clay with loess-like characteristics.

Differing from loess, loess-like silty clay primarily consists of silt and clay particles, with a substantial content of the latter. The soil exhibits weak alkalinity, low solubility of salts, and moderate cation exchange capacity. Similar to loess, particularly under the arid and cold climate conditions of the Yellow River basin, illite as the predominant clay minerals within this soil type, while coarse grains are mainly comprised of detrital minerals such as quartz and feldspar. These characteristics afford the soil adequate water retention and plasticity. Structurally, the pore spaces between the framework units of loess-like silty clay are mainly divided into three types: framework-contact pores, cemented framework-contact pores, and open-framework pores. This diverse pore structure imparts certain compressibility to the soil. However, it is crucial to note that the compression coefficient and liquidity index may vary across different regions, thus requiring meticulous consideration in engineering design.

In summary, our research, conducted through extensive geological surveys and analyses in the middle and lower reaches of the Yellow River, has uncovered the widespread presence of loess-like silty clay, significantly enriching the database on this distinctive soil type and providing crucial references for further study. Loess has been identified as one of the primary sources of this soil type, indicating its potential global occurrence, particularly in loess deposition areas of the middle and lower reaches of rivers. This suggests that our findings are not only applicable within China but also relevant to numerous loess deposition regions worldwide, as evidenced by reports of loess-like silty clay in the Saudi Arabian region. This study will enhance the international community's interest in and understanding of this unique soil type, offering valuable insights for future engineering practices and soil management strategies applicable in a broader global context.

## Data Availability

The datasets generated during and analyzed during the current study are available from the corresponding author on reasonable request.
